# Simple Interventions Can Greatly Improve Clinical Documentation: A Quality Improvement Project of Record Keeping on the Legal Aspect of Dementia at Memory Service

**DOI:** 10.1192/bjo.2022.341

**Published:** 2022-06-20

**Authors:** Shabinabegam Abdul Majid Sheth, Claudia Wald

**Affiliations:** Central and North West London NHS Foundation Trust, London, United Kingdom

## Abstract

**Aims:**

1. To ensure there is documented evidence of discussion about legal aspect of dementia with patients and relatives. 2. To Improve documentation of Financial management by 100% at the end of 3 months. 3.To Improve documentation of LPA and other related legal areas by 70% at the end of 3 months

**Methods:**

In Kensington and Chelsea and Westminster Memory services, we are seeing patients with cognitive impairment which can be due to dementia. So, it is really important to discuss and help a person and/or relative with making decision about client's health, welfare or finances. Though each team member discusses about what they did for the same, we identified that it was not reflected in the documentation.

After finalising data collection form, we then performed a retrospective collection of number of documented assessment/discharge report/progress notes within an identified 3-month time period from March to May 2021. Our intervention was Email and MDT remainders, developing patient information video, and structured format to document about legal aspect of dementia like, financial management, Lasting power of Attorney, Court of protection, Advance will, etc. We run PDSA cycles and collected a data at the end of each month to see whether change ideas are helping to improve documentation and whether any modification will require with the plan.

**Results:**

Out of 67 patients record which were reviewed as a baseline data, 39% of them has diagnosis of dementia or Mild Cognitive impairment. Discussion about LPA was recorded in only 16% of the documents. There is no mention of how a person is managing their finances in 30.7 percent of documents. After implementing change ideas, 29 patients who were seen during the month of December were reviewed using the same data collection form. Documentation of discussion about financial management was improved to 93.1%. Documentation of Discussion about how to set up LPA, if a person doesn't have one has been approved from 10.4% to 55.2%. The cycles will continue for month of January and February and data will be assessed and compared at the end February 2022.

**Conclusion:**

Basis on the data available till date, a general improvement in the record keeping of notes was seen. Simple intervention like email reminders, reminder in MDT, structured format for documentation and patient information in visual format can improve the documentation of work. More detailed conclusion will be drawn at the end of 3 months.

